# Chloroplast DNA Diversity of Tunisian Barley Landraces as Revealed by cpSSRs Molecular Markers and Implication for Conservation Strategies

**DOI:** 10.1155/2022/3905957

**Published:** 2022-09-26

**Authors:** Marwa Snoussi, Leila Riahi, Mériam Ben Romdhane, Ahmed Mliki, Nejia Zoghlami

**Affiliations:** ^1^Laboratory of Plant Molecular Physiology, Centre of Biotechnology of Borj-Cedria, BP 901, Hammam-Lif 2050, Tunisia; ^2^Faculty of Sciences of Bizerte, University of Carthage, 7021 Jarzouna, Bizerte, Tunisia; ^3^Laboratory of Biotechnology and Bio-Geo Resources Valorization BVBGR-LR11ES31, University of Manouba, ISBST, Ariana 2020, Tunisia

## Abstract

In Tunisia, barley local landraces are still cropped for human and animal consumption in some subsistence farming systems under marginal and stressed conditions. These high-value genetic resources present a potential source of resistance genes to biotic and abiotic stresses useful for both national and international breeders. Actually, they are represented by threatened small populations, which face a high risk of genetic erosion and progressive substitution by modern varieties. In this study, the genetic diversity of 60 Tunisian barley landraces was assessed using six chloroplast microsatellites. All loci were found polymorphic, with 2 or 3 alleles per locus. Thirteen alleles were detected across the studied sample, which were combined into 8 haplotypes, giving a haplotype diversity (Hd) of 0.847. High punctual and haplotype genetic diversity was observed for Tunisian barley landraces when compared to other germplasms from other regions of the world. The genetic structure analysis revealed two major clusters of Tunisian barley landraces, which confirms their multiorigin. This result was corroborated by the median-joining network showing the genetic relationships among the eight detected haplotypes. The AMOVA analysis revealed that 83% of the genetic variation is between populations, which requires the *in situ* and *ex situ* conservation of plant material for all Tunisian populations of barley landraces. Information on genetic variation within the chloroplast genome is of great interest to ensure an efficient conservation strategy that takes into account the preservation of the various maternal lineages of Tunisian barley.

## 1. Introduction

Barley (*Hordeum vulgare* L., *Poaceae*) is the fourth main cereal crop, behind only rice, wheat, and corn [1]. It was one of the first cereal species domesticated by humans from its wild relative, *Hordeum spontaneum* K. Koch, in the Fertile Crescent about 10,000 years ago [2]. *Hordeum vulgare* was considered a model species for plant genetic studies for both basic and applied research due to its diploid nature (2*n* = 14), its reduced number of chromosomes, and its ease of crossing [3].

The species has been used for centuries as animal feed, human food, brewing, biodiesel, and for ethanol production [4, 5]. The human consumption of barley grains, which was limited mainly to Asia and Northern Africa, now has an increasing interest in all regions of the world due to its high nutritional value [6]. Indeed, in addition to their richness in dietary fibre, proteins, and minerals, the grains of *H. vulgare* have been shown to be reservoirs of various health-promoting compounds and have gained increasing interest in functional food development [7].

The genetic resources of *H. vulgare* in Tunisia are presented mainly by landraces, which are still cropped for human and animal consumption by some local farmers and are well adapted to marginal and stressed conditions [8]. Barley landraces have a specific value among the genetic resources of this species as they constitute an intermediate evolutionary status between wild and modern barley cultivars and present a reservoir of qualitative and quantitative traits linked to the tolerance of biotic and abiotic stresses [9]. Actually, an increasing interest was attributed to these ancient genetic resources, which were considered a valuable source for the development of new varieties of barley [10]. Barley landraces, which evolved over centuries through the combination of natural and local farmers' selection pressure, showed higher product quality than cultivated germplasms [11].

Despite their interest, the cultivation areas of landraces are limited, and the local farmers face increasing pressure to replace their inherited accessions with introduced commercial varieties considered more homogeneous and yielding [12]. Actually, these high-value germplasms rich in specific useful genes are represented by threatened small populations, which face a high risk of genetic erosion and progressive substitution by modern varieties [9].

The loss of diversity can reduce the capacity of populations to adapt to their changing and stressful environments, and consequently, *in situ* and *ex situ* conservation of local genetic material must be established [13]. Indeed, the occurring climatic changes in Tunisia, as in other regions of the world, manifested by the irregularity of rainfall, high temperatures, and long periods of drought, require the development of new, improved varieties with increased potential to adapt to these changing constraints. Consequently, the valorisation of autochthonous genetic resources, which are already well adapted to local environmental conditions, could open wide horizons for better and sustainable productivity [14].

The characterisation of Tunisian barley's genetic resources was carried out based on morphological descriptors [9], physiological indexes [15], and nuclear DNA molecular markers [16]. However, the cytoplasmic diversity of these valuable germplasms has not yet been explored. Describing the chloroplast DNA genetic diversity is considered a complementary tool to molecular surveys of the nuclear genome and provides relevant information for genetic analyses and conservation issues in cereal crops [14].

Given their uniparental maternal mode of inheritance in angiosperms, chloroplast molecular markers present an efficient tool to infer the parent ancestry in putative hybrid progeny [17] and were applied to discriminate among the sterile and fertile plant germplasms, which help genomic selection [18]. Simple sequence repeats (SSRs) are the molecular markers of choice for chloroplast genome genotyping. These molecular markers have been used to evaluate the status of genetic diversity and relationships in cultivated and wild germplasms of barley [19, 20]. Moreover, cpSSRs were applied to support the polyphyletic origin of barley [21]. In other reports, cpSSRs have shown their ability to clarify the molecular evolution pattern, the origin of barley genetic resources, and to give information concerning the occurrence of new domestication events for this species [22].

Based on the last considerations, the present study focused on the molecular characterisation of 60 accessions representing eleven populations of Tunisian barley landraces using 6 cpSSR (chloroplast simple sequence repeats) molecular markers. The genotyped genetic resources are representative of the main distribution areas of this crop in Tunisia.

## 2. Materials and Methods

### 2.1. Plant Material and DNA Extraction

The seeds of 60 accessions of Tunisian local barley representing 11 landraces populations were collected from different regions belonging to various bioclimatic stages to serve as plant material for this study. The bioclimatic characteristics of the investigated regions are described in Table 1. Further details concerning the sampling of the studied genetic resources of barley are reported by Ben Romdhane et al. [16]. Chloroplast genomic DNA was extracted using the CTAB method [23] as optimized by Zoghlami et al. [9] from two-week-old seedlings germinated under semicontrolled conditions. For each accession, the DNA was extracted by pooling the plant material of 10 germinated seeds.

### 2.2. Molecular Analysis

Molecular analysis of the chloroplast genome of 60 Tunisian barley accessions was carried out using 6 cpSSR markers (Table 2), previously developed from the barley genome [19]. PCR amplifications were performed as described by Riahi et al. [14] in a total volume of 10 *μ*l as follows: 1 *μ*l PCR buffer, 0.2 *μ*l DNTPs (25 mM), 0.7 *μ*l MgCl_2_ (25 mM), 1 *μ*l DNA (10 *μ*M), 1 *μ*l primer F and 1 *μ*l primer R (10 *μ*M), 0.1 *μ*l Taq polymerase (0.5 U), and 5 *μ*l of distilled water. The amplified DNA fragments were separated by electrophoresis using a 2.5% agarose gel. A 100-bp DNA ladder was used to determine the allele's size.

### 2.3. Data Analysis

Various allelic and haplotype genetic parameters were estimated across the 60 barley landraces. The software GenAlEx 6.5 [24] was used to estimate the number of alleles per locus (Na), the number of effective alleles per locus (Ne), the Shannon index (I), the genetic diversity (h), and the unbiased genetic diversity (Uh). The numbers of the detected haplotypes (A), the frequencies and the distribution of haplotypes, the numbers of private haplotypes (P), the effective number of haplotypes (Ne), and the haplotypic diversity (Hd) were determined using the haplotype analysis software v.1.05 [25].

The genetic relationships and genetic structure analyses among the studied barley populations were assessed. The UPGMA analysis was carried out using Darwin software v.6.0.021, applying dissimilarity indexes calculated from single data [26]. Molecular variance analysis (AMOVA) between and within populations along with genetic differentiation (PhiPT) with 999 permutations was achieved using the GenAlEx 6.5 program [24]. The genetic relationships among the detected chloroplast haplotypes were highlighted based on a median-joining network using the software network 4.5.1.6 [27].

## 3. Results

In this study, 6 cpSSR loci were used to assess the chloroplast genetic diversity of 60 local Tunisian barley accessions representing the distribution area of this species in Tunisia. The obtained results showed that the loci are all polymorphic. The genotyping of the total sample allowed the detection of 13 alleles with a mean value of 2.167 ± 0.167 alleles per locus. The effective number of alleles per locus varies from 1.220 for the locus hvcptrnS1 to 2.410 for hvcptrnS2, with a mean value of 1.814 ± 0.165. Shannon's information index varies from 0.325 to 0.960 with a mean value of 0.637 ± 0.084. The genetic diversity indices h and uh vary from 0.180 and 0.183 (hvcptrnS1) to 0.585 and 0.595 (hvcptrnS2), respectively (Table 2).

Due to the haploid nature of chloroplast DNA, a unique assembly between the six genotyped microsatellite loci was considered a distinct haplotype. The thirteen identified alleles at the 6 loci were combined into 8 haplotypes with haplotypic frequencies that range between 0.050 for haplotype 8 and 0.280 for haplotype 7 (Table 3). A haplotype genetic diversity (Hd) value of 0.847 was recorded for the total analysed sample of 60 barley landraces. The distributions of the 8 detected haplotypes across the investigated populations along with their frequencies are shown in Table 3.

It is noted that one unique haplotype was detected for each of the five barley populations Beja (haplo-3), Bizerte (haplo-1), Gabes (haplo-7), Kasserine (haplo-8), Kef (haplo-6), and Zaghouan (haplo-2), while two haplotypes were observed for each of the remains populations (haplo-2 and haplo-7 for the populations of Mahdia, Sfax, and Sousse; haplo-4 and haplo-7 for Medenine; and haplo-5 and haplo-8 for Siliana). One private haplotype was observed for each of the populations Beja (haplo-3), Bizerte (haplo-1), Kef (haplo-6), Medenine (haplo-4), and Siliana (haplo-5). The effective number of haplotypes (Ne) ranged between 1 (Beja, Bizerte, Gabes, Kasserine, Kef, and Zaghouan) and 2 haplotypes (Sousse). The haplotype genetic diversity (Hd) varied among the 11 populations between 0 and 1, with a mean value of 0.260 (Table 4).

**Figure 1:**
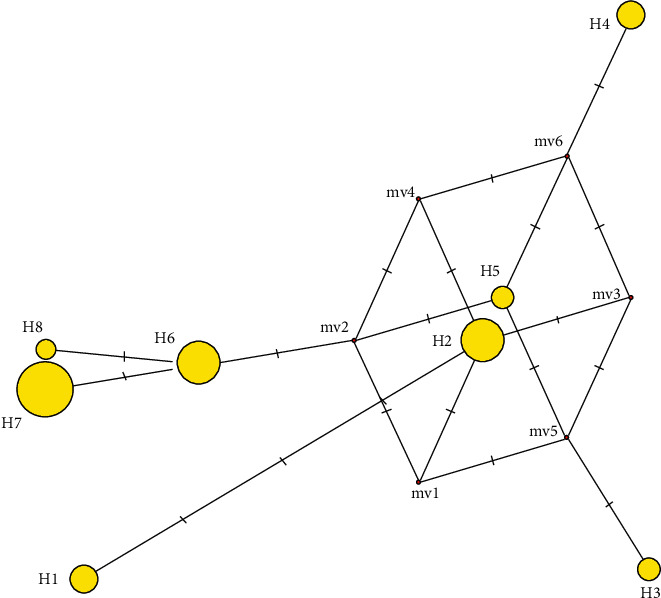
Median-joining network showing the genetic relationships among the eight detected chloroplast haplotypes. Circle sizes are proportional to the haplotype frequencies in the total studied landraces.

In order to clarify the genetic relationships among the 8 detected haplotypes (H) in Tunisian barley landraces, a median-joining network was constructed (Figure 1). This analysis revealed that the most frequent haplotype, H7, is found to be genetically related to the haplotypes H8 and H6. The haplotype H2 occupies a central position, while a genetic distinction of the haplotype 1 was revealed by this analysis.

**Figure 2:**
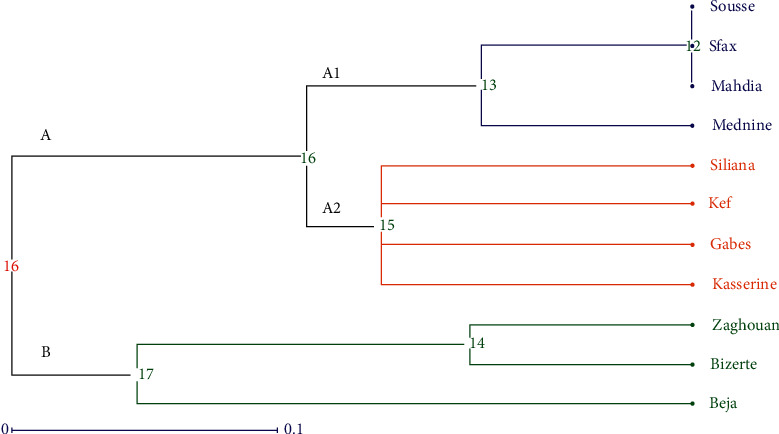
UPGMA dendrogram representing the genetic relationships between the 11 studied populations of barley landraces based on six cpSSR (chloroplast microsatellites) molecular markers.

An UPGMA dendrogram was constructed to evaluate the genetic relationships between the 11 studied populations of Tunisian barley landraces (Figure 2). The obtained dendrogram showed clearly the classification of Tunisian germplasm into two major clusters. The first cluster, A, is divided into 2 subclusters, (A1) and (A2). The subcluster (A1) regroups two populations of the Centre-East (Sousse and Mahdia) and two populations of the South-East region (Sfax and Medenine), while the populations of Gabes, Siliana, Kef, and Kasserine were classified in the subcluster A2. The second cluster (B) contains the accessions of Zaghouan, Beja, and Bizerte. A genetic structure based on the geographical origin of the studied populations was observed, with some overlap between the studied barley populations.

The analysis of molecular variance (AMOVA) was achieved to analyze the amounts of genetic diversity between and within populations (Table 5). Based on this analysis, the majority of the observed genetic variation was attributed to the differences between the analysed populations (83%), while a smaller amount of genetic diversity (17%) was attributed to the differences within populations. The genetic differentiation analyses confirmed these findings and showed highly significant genetic differentiation values (PhiPT = 0.828, *P* value = 0.001) among the local barley populations.

## 4. Discussion

The molecular characterisation of Tunisian barley landraces showed that the six used loci are all polymorphic across the genotyped barley sample and allowed the detection of 13 total alleles. Two to three alleles per locus were detected, with a mean value of 2.16 alleles per locus. The detected polymorphism in this study is considered as important given the haploid nature of the studied loci and the mating system of barley. The obtained results corroborate previous studies, which focused on chloroplast SSRs diversity in the barley genome and showed that two to three alleles per locus were detected [19, 21, 28]. The low detected number of alleles per locus for barley cpSSR molecular markers is explained by a high conservation level of the chloroplast genome. Due to the absence of heteroplasmy and recombination, angiosperm species have highly conserved their chloroplast genomes, which result in a low evolution rate compared to nuclear genomes [29].

A total of 8 haplotypes were identified in Tunisian barley landraces based on the six cpSSR loci. The analysis of the haplotype diversity among the studied populations showed that the number of haplotypes per population varies between one and two haplotypes. A high haplotypic genetic diversity (0.847) across the studied 60 landraces is detected. This level is higher than values previously reported for other studies interested in barley landraces. A haplotype genetic diversity of 0.330 was recorded for Turkish barley landraces [28], while Provan et al. [19] reported a value of 0.471 for barley landraces originated from Syria and Jordan.

The detected variation in the amounts of haplotype diversity among the studied samples of barley landraces may be explained by various factors, namely the number of analysed landraces, the geographic and bioclimatic characteristics of the prospected areas, and the domestication status of the analysed plant material. Indeed, the domestication process of barley from its wild progenitors resulted in a dramatic reduction in its genetic diversity. Lower genetic diversity amounts for barley landraces compared to wild accessions and for cultivars compared to landraces were previously highlighted by applying chloroplast molecular markers [21]. Thus, the genetic diversity of landraces is expected to decrease with the increase of selection practices through the cropping cycles, which tends towards their homogenization. The evolution of Tunisian barley landraces throughout thousands of years under contrasting and extreme edaphic and bioclimatic conditions may explain their considerable genetic diversity and high adaptation potential. On the other hand, given the geographical location and historical facts of Tunisia, the enrichment of the local barley germplasm by introducing plant material of this species during the settlement of many civilizations is not excluded and may explain in part the considerable genetic diversity detected for Tunisian cereal crops [10, 14].

The genetic structure analysis of the studied germplasms revealed two main clusters. Two main clusters were also obtained when five barley cpSSRs were applied to investigate the genetic relationships among 186 barley accessions representing cultivated and wild germplasms that originated from the main distribution areas of this crop except for the Far East region [21]. The observed pattern of genetic relationships among Tunisian barley landraces revealed a low genetic structure according to the geographical origins of the studied populations. This corroborates the obtained results applying nuclear SSR molecular markers and morphological descriptors for Tunisian barley landraces and is explained by the seeds exchange that occurred between farmers belonging to some cropping regions [9, 16].

The phylogenetic relationships among the eight detected haplotypes evaluated by a median-joining network revealed a complex genetic relationship pattern. This analysis highlighted three frequent haplotypes and other minor haplotypes that may have evolved through mutation events in these major haplotypes over time. Indeed, the most frequent and shared haplotypes are generally considered to be the most ancient ones, and minor haplotypes for chloroplast SSRs appeared generally following the stepwise mutation process [30].

The obtained results showed considerable allelic and haplotype genetic diversity for the chloroplast genome of Tunisian barley landraces. The detected molecular polymorphism in these threatened local landraces may be lost over time if any conservation measures are not achieved. The conservation of the genetic resources of barley, especially ancient landraces, is required to maintain the maximum allelic richness that can be exploited in the future to enhance the quality and yields of this species [16]. Indeed, breeding strategies rely on the genetic variation of these valuable genetic resources [11].

The AMOVA analysis of the studied landraces revealed that 83% of the observed genetic variability is among populations. For agricultural crops, human selection and management in addition to natural selection are considered the main factors that define the patterns of intraspecific genetic diversity and differentiation [31]. The observed genetic differentiation pattern in this investigation gives high conservation priorities for all the remaining populations of Tunisian barley landraces.

Chloroplast genomes were reported to contain genes relevant to the expression of various agronomically important traits such as cytoplasmic male sterility, plant growth, and response to adverse conditions [32–34]. Thus, the *ex situ* conservation in gene banks of the seeds of all the detected maternal lineages is of great interest for a further improvement strategy for barley. Furthermore, *in situ* conservation measures are required as a complementary conservation strategy to allow the evolution of these valuable landraces in the various edaphic and bioclimatic conditions of Tunisia to maintain their genetic polymorphism. It is necessary to pay specific attention to the conservation of landraces with private and rare alleles and haplotypes.

While the challenge of the *ex situ* conservation of local germplasms of cereal crops, including barley landraces, has been started over the last decade in Tunisia, more urgent efforts are needed. In addition to the static *ex situ* conservation tool, the establishment of dynamic *in situ* collections of barley landraces with various edaphic and bioclimatic characteristics is required to ensure the preservation and evolution of their adaptation potentials in the face of changing environmental conditions.

## 5. Conclusions

The present study illustrates the efficiency of a set of informative chloroplast sequence repeats (cpSSRs) to assess the variation of genetic diversity, structure, and differentiation among various gene pools of barley landraces. A high haplotype genetic diversity was detected for Tunisian barley landraces, which are clustered into two main groups. These cpSSRs present complementary molecular tools to nuclear molecular markers to evaluate the maternal lineage diversity and classification of barley landraces. The characterisation of the cytoplasmic genome of Tunisian local barley landraces is of great interest for the establishment of conservation strategies for this cereal crop, which will be useful as a source of specific traits in future breeding programs.

## Figures and Tables

**Table 1:** Characteristics of the studied populations of Tunisian barley landraces.

Population	Sample size	Region of Tunisia	Bioclimatic stage
Beja	4	North West	Semiarid-Subhumid
Kef	10	North West	Semiarid-Subhumid
Siliana	5	North West	Semiarid-Subhumid
Zaghouan	5	North East	Semiarid-Subhumid
Bizerte	6	North East	Semiarid-Subhumid
Kasserine	2	Centre West	Arid superior
Sousse	2	Centre East	Semiarid
Mahdia	5	Centre East	Arid superior-Semi arid
Sfax	8	South East	Arid inferior-Arid superior
Gabes	5	South East	Arid inferior
Medenine	8	South East	Arid inferior

**Table 2:** Characteristics of the six cpSSR (chloroplast microsatellites) molecular markers and genetic diversity parameters across the 60 studied barley landraces. Tm: primer melting temperature, Na: number of alleles per locus, Ne: number of effective alleles per locus, I : Shannon's information index, h: genetic diversity, uh: unbiased genetic diversity.

Locus	Repeats	Tm°C	Na	Ne	*I*	*h*	Uh
hvcppsbA	(T)_8_	50	2	1.800	0.637	0.444	0.452
hvcprpoA	(T)_8_(CTT)_3_	60	2	2.000	0.693	0.500	0.508
hvcprps12	(T)_8_	60	2	1.557	0.543	0.358	0.364
hvcptrnS1	(A)_7_CGC(T)_13_	60	2	1.220	0.325	0.180	0.183
hvcptrnS2	(T)_10_	60	3	2.410	0.960	0.585	0.595
hvcptrnLF	(C)_9_	60	2	1.897	0.666	0.473	0.481
Means			2.167	1.814	0.637	0.423	0.431
SD			0.167	0.165	0.084	0.057	0.058

**Table 3:** Distribution and frequencies of the detected chloroplast haplotypes among the investigated populations of barley landraces.

Haplotype code	Total sample	Beja	Bizerte	Gabes	Kasserine	Kef	Mahdia	Medenine	Sfax	Siliana	Sousse	Zaghouan
Haplo-1	0.10	0.00	1.00	0.00	0.00	0.00	0.00	0.00	0.00	0.00	0.00	0.00
Haplo-2	0.17	0.00	0.00	0.00	0.00	0.00	0.40	0.00	0.25	0.00	0.50	1.00
Haplo-3	0.07	1.00	0.00	0.00	0.00	0.00	0.00	0.00	0.00	0.00	0.00	0.00
Haplo-4	0.10	0.00	0.00	0.00	0.00	0.00	0.00	0.75	0.00	0.00	0.00	0.00
Haplo-5	0.07	0.00	0.00	0.00	0.00	0.00	0.00	0.00	0.00	0.80	0.00	0.00
Haplo-6	0.17	0.00	0.00	0.00	0.00	1.00	0.00	0.00	0.00	0.00	0.00	0.00
Haplo-7	0.28	0.00	0.00	1.00	0.00	0.00	0.60	0.25	0.75	0.00	0.50	0.00
Haplo-8	0.05	0.00	0.00	0.00	1.00	0.00	0.00	0.00	0.00	0.20	0.00	0.00

**Table 4:** Variability of the haplotype genetic indices among the studied populations. A : number of detected haplotypes, P : number of private haplotypes, Ne : effective number of haplotypes, Hd : haplotype genetic diversity.

Population	A	*P*	Ne	Hd
Beja	1	1	1.000	0.000
Bizerte	1	1	1.000	0.000
Gabes	1	0	1.000	0.000
Kasserine	1	0	1.000	0.000
Kef	1	1	1.000	0.000
Mahdia	2	0	1.923	0.600
Medenine	2	1	1.600	0.429
Sfax	2	0	1.600	0.429
Siliana	2	1	1.471	0.400
Sousse	2	0	2.000	1.000
Zaghouan	1	0	1.000	0.000
Means	**1.455**	**0.455**	**1.327**	**0.260**

**Table 5:** Analysis of molecular variance (AMOVA) between the 11 populations of Tunisian barley landraces applying six cpSSR (chloroplast microsatellites) molecular markers.

Source	Df	SS	MS	Est. Var.	%	PhiPT	*P* Value
Among populations	10	107.400	10.740	1.931	83	0.828	0.001
Within populations	49	19.700	0.402	0.402	17		
Total	59	127.100		2.333	100		

SS: sum of squares, MS: mean square, Est. Var: estimated variance.

## Data Availability

The datasets used to support the findings of this study are included within the article and are available from the corresponding author upon request.
